# Loss of IL13RA2 promotes metastatic tumor growth in triple-negative breast cancer via increased AKT and NF-κB signaling

**DOI:** 10.1007/s10585-025-10362-1

**Published:** 2025-07-15

**Authors:** Wendy E. Bindeman, Kevin C. Corn, Marjan Rafat, Barbara Fingleton

**Affiliations:** 1 Program in Cancer Biology, Vanderbilt University, Nashville, TN USA; 2 Department of Chemical and Biomolecular Engineering, Vanderbilt University, Nashville, TN USA; 3 Department of Biomedical Engineering, Vanderbilt University, Nashville, TN USA; 4 Department of Radiation Oncology, Vanderbilt University, Nashville, TN USA; 5 Department of Pharmacology, Vanderbilt University, 2220 Pierce Ave, 771 PRB, Nashville, TN 37232 USA

**Keywords:** Brain metastasis, Triple-negative breast cancer, IL13RA2, Signaling, AKT, NF-κB

## Abstract

Triple-negative breast cancer is associated with poor patient prognosis and high rates of distant metastasis. These patients are at elevated risk of brain metastasis, which remains a major therapeutic challenge. IL13RA2, a high-affinity receptor for IL13, is highly expressed in primary brain cancers, many extracranial solid tumors, and in lung- and brain-seeking metastatic variant cell lines. However, the relationship between IL13RA2 and patient prognosis is variable, and the biological function of this receptor in cancer remains controversial. We sought to define the role of IL13RA2 in triple-negative breast cancer growth and metastasis, with an emphasis on breast-to-brain metastasis. We generated IL13RA2-CRISPR knockout derivatives of the human brain-seeking breast cancer cell line MDA231BrM2, as well as murine 4T1 cells, and evaluated changes in gene expression, proliferation, survival, and metastatic growth in vivo. Both IL13RA2-deficient models demonstrate enhanced cell survival in vitro, as well as augmented metastatic tumor growth and worsened animal survival in intracardiac models of brain metastasis. Concordantly, elevated *IL13RA2* mRNA expression is positively correlated with overall survival in patients with basal-like breast cancer. Mechanistically, IL13RA2-deficient cells exhibit increased AKT and NF-κB signaling. These cells are sensitive to inhibition of either pathway, but especially AKT, which may represent a clinically useful vulnerability for patients with IL13RA2-low tumors. Our data suggest that inhibition of IL13RA2, though promising in other tumor contexts, may be deleterious in metastatic triple-negative breast cancer.

**Supplementary Information:**

The online version contains supplementary material available at 10.1007/s10585-025-10362-1.

## Introduction

Triple-negative breast cancer (TNBC), characterized by lack of expression of estrogen receptor, progesterone receptor, and HER2, accounts for approximately 11% of female breast cancer diagnoses [1]. Basal-like breast cancer is similar but defined by transcript rather than protein expression [2]. Due to lack of targeted therapies and the aggressive disease course, TNBC prognosis is frequently poor and improved treatment options are warranted.

Metastasis is a major factor in cancer morbidity and mortality. Even when diagnosed at an early stage, TNBC patients have a high risk of recurrence and distant metastasis; 30–50% will develop distant metastases [3, 4]. Brain metastases (BrM) are the most frequently diagnosed intracranial tumor in the United States [5] and rates of diagnosis are rising, due to improved screening practices and imaging [6, 7], as well as extended patient survival as therapies improve [6–8]. Patients with TNBC are at high risk for brain involvement, with up to 40% developing BrM [9]. Current treatments are ineffective and cause neurocognitive side-effects [10]. Thus, it is imperative to understand drivers of brain metastasis and develop effective therapies.

IL13RA2, a high-affinity receptor for interleukin (IL) 13 (IL13), is only expressed in the testes under normal physiological conditions. However, it is present at high levels in malignant gliomas and some solid tumors [11, 12] and is therefore considered a cancer/testis antigen [13]. IL13RA2 is highly expressed by brain- and lung-seeking breast cancer cell lines [14, 15] and is often reported to be pro-tumorigenic, pro-metastatic, and correlated with poor patient survival [16–22]. However, other reports suggest that elevated IL13RA2 in tumor cells [23–25] or cancer-associated fibroblasts [26] correlates with improved patient survival. Further, overexpression of IL13RA2 inhibits breast and pancreatic tumor growth in vivo [27], and silencing of the receptor promotes proliferation, survival, and migration in hepatocellular carcinoma [24].

IL13RA2 was originally identified in relation to IL13RA1 [28, 29], a component of the type II IL4 receptor (type II IL4R) system [30]. Type II IL4R is expressed on epithelial cancer cells, including breast cancer, responds to stimulation by either IL4 or IL13, and its elevated expression is associated with breast cancer metastasis [30–33]. Both IL4 and IL13 have been shown to be elevated in various cancer types [30, 34]. IL13RA2 was originally thought to lack signaling activity and function solely as a decoy receptor [35] whose main function was to limit type II IL4R activation by IL13 [36]. More recent work has identified diverse functions for IL13RA2 independent of its interplay with type II IL4R, including IL13-activated AP-1 [37–40] and Src-related pathways [18–21], and, when complexed with TMEM219, chitinase-3 like protein 1 (CHI3L1)-activated signaling via MAP kinase, AKT/PKB, and WNT [41, 42].

There is substantial interest in exploiting tumor-specific IL13RA2 for either payload targeting or direct inhibition [43–46]. Given the complex and context-dependent roles of IL13RA2, it is important to understand its biological role within a specific tumor type before manipulating its activity. This study aims to elucidate IL13RA2 function in TNBC. We show that IL13RA2 deletion promotes cancer cell proliferation and survival in vitro and enhances tumor cell metastasis in vivo. AKT and NF-κB signaling are elevated in IL13RA2-deficient cells, resulting in increased sensitivity to inhibition of both pathways. Overall, our work suggests that in metastatic TNBC, inhibition of IL13RA2 may be deleterious to patients, though patients with IL13RA2-low tumor may benefit from AKT inhibitors.

## Methods

*Reagents*: Detailed information regarding sources, catalog numbers and version information for antibodies, key biochemical reagents, and software mentioned below is provided in Online Resource 1 (Table S1).

*Cell culture*: All cells were maintained in Dulbecco’s Modified Eagle’s Medium (DMEM) supplemented with 10% fetal bovine serum (FBS) and 50 µg/mL gentamycin sulfate in a humidified incubator at 37 °C and 5% CO_2_. Cells were routinely tested for mycoplasma. None of the cell lines used are reported as misidentified (ICLAC.org; accessed 11 February 2025).

MDA 231-BrM2-831 (GFP+/luciferase+) cells were from the Memorial Sloan Kettering Cancer Center Antibody and Bioresource Core Facility (RRID: SCR_017691) (New York, NY). The cell line has been authenticated by short tandem repeat (STR) analysis. MDA-MB-231 and unmodified 4T1 (4T1-WT) cells were purchased from American Type Culture Collection (ATCC). Luciferase-expressing 4T1 cells (4T1-luc) were previously described [31].

*Generation of ΔIL13RA2 cell lines*: All-in-one CRISPR plasmids (pCRISPR-CG08) containing guide RNAs for human or murine IL13RA2, Cas9 and a fluorescent marker were obtained from Genecoepia. 4T1-luc or MDA231BrM2 cells were transfected with CRISPR plasmids containing three IL13RA2-targeting guide RNAs or scramble control using Lipofectamine 3000. Successfully deleted cells were isolated by sorting for the fluorescent marker, followed by sorting for IL13RA2-negative cells on a BD FACSAria III performed by the Vanderbilt Flow Cytometry Shared Resource (FCSR). Due to lack of an effective antibody for murine Il13ra2, the second sort was not possible for 4T1-ΔIl13ra2.

*RNA extraction and qPCR*: Cells were plated and incubated in DMEM + 2% FBS (2% media) ± IL4/IL13 as indicated for 48 h before lysis in TRIzol. Brain tissue from mice was flash-frozen immediately following dissection and dissociated in TRIzol. RNA was isolated using the Direct-zol RNA Miniprep kit. cDNA was made using the High Capacity cDNA kit. Quantitative real-time PCR (qPCR) was performed using PowerUP SYBR Green Master Mix. Primer information is provided in Online Resource 2 (Table S2). All qPCR was run on a QuantStudio3 machine. Data are expressed as either fold change relative to housekeeping gene (2^−ΔCt^) or relative to control (2^−ΔΔCt^) as indicated.

*Signaling evaluation*: Cells were seeded in DMEM + 10% FBS and adhered overnight before switching to serum-free DMEM 18–24 h prior to addition of species-specific cytokines at indicated concentrations for 20–30 min.

*Conditioned media*: Cells were plated in 2% media (with IL13 for 4T1) and incubated for 3 days to generate conditioned media (CM). Receiver plates of MDA-MB-231 or 4T1-CTL were seeded in DMEM + 10% FBS for 2 days and subsequently serum-starved, incubated with CM for 10 min, followed by a 10 min pulse with IL13, and lysates collected.

*Lysate preparation and Western blotting*: As previously described [33], lysates were prepared, analyzed for protein content, and electrophoresed. Gels were then transferred to nitrocellulose membranes and blocked prior to overnight incubation with primary antibodies at 4 °C. Incubation with secondary antibodies, chemiluminescent detection, stripping and re-probing was performed as previously described [33]. Band intensity was measured using Fiji ImageJ [47].

*Flow cytometry*: Cells were dissociated from culture plates and incubated with PE-anti human IL13RA2 for 20 min. Subsequently, samples were stained with DAPI and run on a BD LSRFortessa instrument available for independent use through the FCSR. Data were analyzed using FlowJo software.

*Cell cycle analysis*: Cells were seeded at 1–2 × 10^5^cells/well in 2% media and incubated for 72 h ± species-specific IL4 or IL13, ipatasertib, BMS-345,541, or vehicle control (DMSO) as indicated, prior to 1 h treatment with EdU. Cells were dissociated, stained with Zombie NIR viability dye, fixed in 4% paraformaldehyde, and permeabilized using 0.1% Triton-X. Samples were incubated with 2 mM CuSO_4_, 8 µM AF 568 Azide, and 20 mg/mL ascorbic acid for 30 min, and stained with Hoechst before measurement on a BD LSRFortessa instrument, available for independent use through FCSR. Data were analyzed using FlowJo.

*Cell survival analysis*: Cells were seeded at 1–2 × 10^5^cells/well in 2% media and incubated for 72 h ± species-specific IL4 or IL13, ipatasertib, BMS-345,541, or vehicle control (DMSO) as indicated. Cells were stained with ApoTracker Tetra (AF647) and DAPI and measured/analyzed using the BD LSRFortessa flow cytometer as described for cell cycle analysis.

*Cyquant assay*: Cells were seeded in 2% media at 1 × 10^4^ cells/well in 96-well plates and incubated overnight. Ipatasertib, BMS-345541, or vehicle control (DMSO) were each diluted in 2% media and added to replicate wells (8–16/treatment) to the final concentrations noted in figure legends. After 72 h incubation, Cyquant Direct reagent was prepared according to manufacturer’s instructions and added to wells. The plates were read 1 h later using an Odyssey M instrument.

*Clonogenic assay*: 4T1 derivatives were seeded at 200 cells/well in 24-well plates. For inhibitor experiments, cells were allowed to attach overnight and subsequently treated with ipatasertib or BMS-345541 in 2% media with re-treatment after 3 days. After 6 days, colonies were stained in 0.5% crystal violet/6% glutaraldehyde and quantified using the GelCount System available through the Digital Histology Shared Resource (DHSR).

*In vivo experiments*: 6–8-week-old female Nude (Nu: J) or BALB/c mice were purchased from Jackson Labs (Bar Harbor, ME) and acclimated for one week before use. All mice were housed in Vanderbilt’s accredited animal facility and studies were approved by Vanderbilt’s Institutional Animal Care and Use Committee (protocol M2300038-00). Body condition and weight were monitored twice weekly.

*Intracardiac injection and bioluminescent imaging (BLI)*: 1–3 × 10^5^ MDA231BrM2 or 4T1 derivatives were administered to nude or BALB/c mice, respectively, by intracardiac injection. Successful cell delivery to the brain was confirmed by BLI imaging 4–24 h after injection and tumor development monitored 1–2x weekly on an IVIS Spectrum instrument as previously described [48]. Images were analyzed using Living Image software. Animals lacking head signal at initial imaging were excluded.

*Tissue histology*: Mouse brains were formalin-fixed, paraffin-embedded, sectioned, and stained with hematoxylin and eosin (H&E). Sectioning was performed in the Tissue Pathology Shared Resource (TPSR), and images of H&E-stained sections were obtained by DHSR using an Aperio Versa 200 instrument. The images were stored on the DHSR’s digital slide archive and downloaded as JPEG files (due to file size limitations) using the Histomics user interface (Kitware.com). To quantify percent tumor area, slide images were analyzed using Fiji ImageJ [45]. Images were deconvoluted using the H&E preset vector. The eosin binary image was used to measure the total tumor section area by thresholding to area of positive staining, and the hematoxylin binary image was used to measure area of tumor nodules.

*Immunohistochemistry*: Slides were deparaffinized, rehydrated, treated with hydrogen peroxide to deactivate endogenous peroxidases before citrate (pH 6.0)-mediated antigen retrieval, blocking and overnight incubation with primary antibody as previously described [31]. After washing, incubation with a biotinylated secondary antibody and amplification using the Vector Laboratories ABC system according to manufacturer’s instructions, slides were incubated with DAB as previously described [31]. Slides were then counterstained using hematoxylin, rinsed, dehydrated through graded alcohols followed by xylenes and cover-slipped. Slide images were captured using an Olympus BX41 microscope and CellSens software with 5 high-power fields randomly captured per slide. Quantification was performed using the count tool in Adobe Photoshop.

*RNA-Seq*: 10^5^ cells/well were plated in 2% media for 48 h. 4T1 were treated with IL13 and RNA was isolated as described above. Bulk RNA sequencing and all analysis was performed by Novogene Corporation Inc. GSEA heatmaps are reproduced from diagrams created by Novogene.

*Statistics*: All data displayed as mean ± SEM. All qPCR experiments were performed in technical triplicate. Unless otherwise indicated, qPCR results are reported as compiled data from independent experiments normalized to the average of the appropriate controls. The number of independent repeats for each experiment is indicated in figure legends. Sample sizes for in vivo experiments are also indicated in figure legends. Patient data in Fig. S2a (Online Resource 3) obtained from KM Plotter [49] (https://kmplot.com), accessed 28 January 2025. Patient data in Fig. S2b (Online Resource 3) obtained from MBCProject.org via the CBioPortal interface (https://cbioportal.org), accessed 15 June 2025. Differences between experimental groups were calculated using t-tests, Mann-Whitney, Kruskal-Wallis with Dunn’s multiple comparisons, one-way ANOVAs with Šídák’s multiple comparisons, or Log-rank (Mantel-Cox) tests as appropriate. Statistical analysis, including reanalysis of KM Plotter dataset, performed in GraphPad Prism.

## Results

### IL13RA2 is highly elevated in brain-seeking TNBC cells

**Fig. 1:**
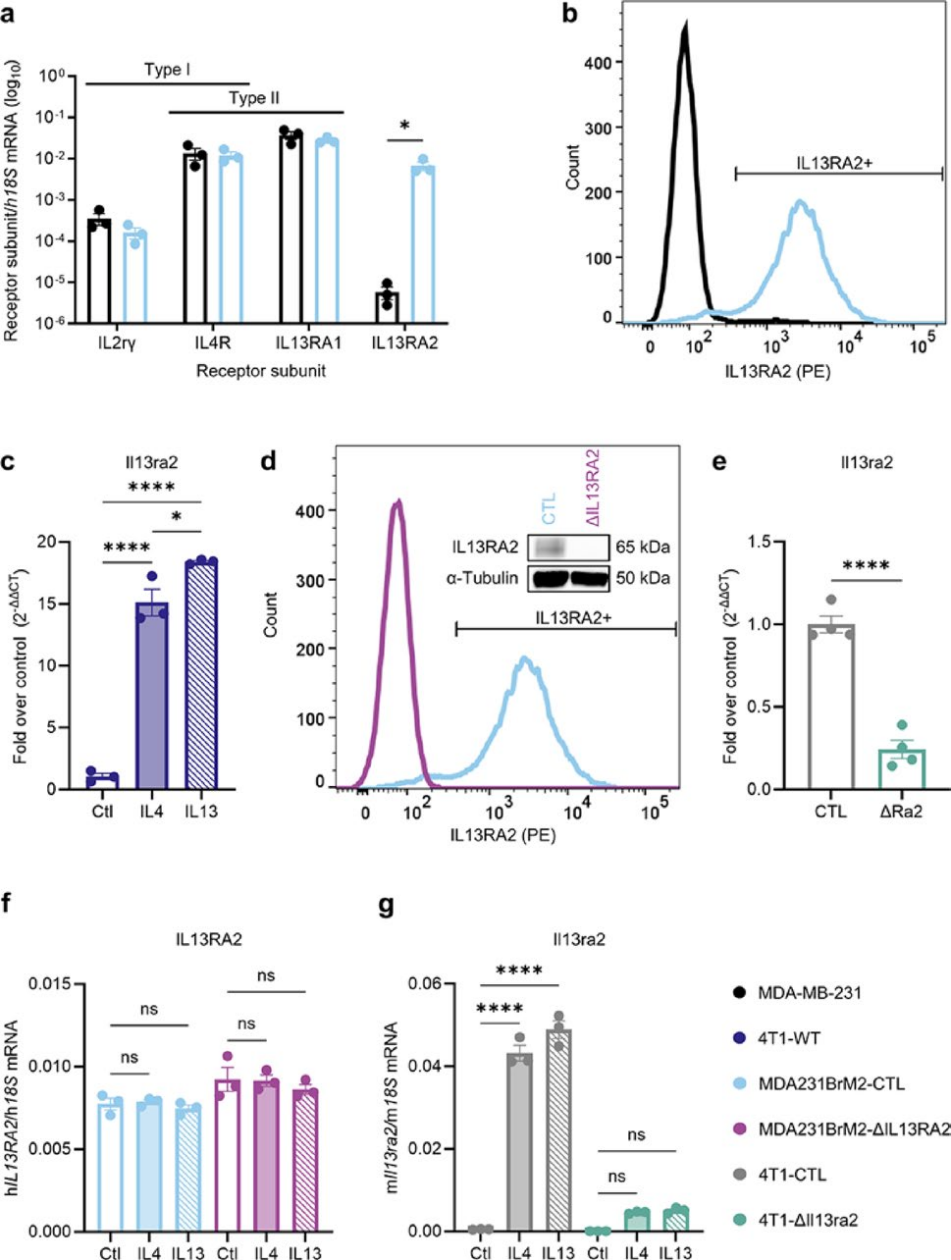
IL13RA2 is elevated in brain-seeking TNBC cells, conditionally expressed in a murine model of TNBC, and can be successfully deleted from both cell lines. **a** Comparison of transcript levels of *IL13RA2*, type I IL4 receptor components, and type II IL4 receptor components in MDA231BrM2-CTL, a brain-seeking TNBC line, and non-brain-seeking MDA-MB-231. Data shown as unnormalized averages from three separate experiments. Statistical significance was determined by an unpaired t-test with *p < 0.05 (*n* = 3). Non-significant comparisons omitted for clarity. **b** Comparison of IL13RA2 protein levels in MDA231BrM2-CTL (IL13RA2+: 92.6%) and MDA-MB-231 (IL13RA2+: 2.83%). Representative data (*n* = 4). **c** Comparison of *Il13ra2* transcript levels in wild-type 4T1 (4T1-WT) cells following stimulation with IL4 or IL13. Representative data from three internal biological replicates shown (*n* = 1 independent experiment in 4T1-WT; phenotype re-confirmed in 4T1-CTL, see (**f**)). **d** Validation of IL13RA2 deletion in MDA231BrM2-ΔIL13RA2 by Western blot (inset, *n* = 2) and flow cytometry (*n* = 3). IL13RA2+: 92.6% in MDA231BrM2-CTL; 0.060% in MDA231BrM2-ΔIL13RA2. **e** Validation of *Il13ra2* deletion in 4T1-ΔIl13ra2 by qPCR. Statistical significance was determined by an unpaired t-test with ****p < 0.0001 (*n* = 4). **f** Evaluation of *IL13RA2* transcript levels in MDA231BrM2-CTL and MDA231BrM2–ΔIL13RA2 following IL4/IL13 stimulation. **g** Evaluation of *Il13ra2* transcript levels in 4T1-CTL vs. 4T1-ΔIl13ra2 following IL4/IL13 stimulation. **f, g** Representative data shown (*n* = 3 independent experiments). IL4, 10ng/mL; IL13, 20ng/mL, 48 h. Statistical significance was determined by one-way ANOVA with Šídák’s multiple comparisons test with ****p < 0.0001 and ns, non-significant (*p* > 0.05). All error bars show SEM

Given the overexpression of IL13RA2 in multiple cancer types, and its previous identification as highly expressed in brain-tropic breast cancer cells [14], we sought to investigate its role in breast cancer brain metastasis. MDA 231-BrM2-831 (referred to as MDA231BrM2) is a brain-seeking derivative of MDA-MB-231 developed by the Massagué lab [14]. By qPCR analysis, these cells express *IL13RA2* at highly elevated levels compared to non-brain-seeking MDA-MB-231 (Fig. 1a). Concordantly, MDA231BrM2 express high protein levels of IL13RA2, while MDA-MB-231 lack the receptor (Fig. 1b). We chose 4T1, a murine model of TNBC, as a secondary cell line model. 4T1 is well-established as a highly aggressive, syngeneic TNBC model; even unselected 4T1 cells metastasize to the brain following intracardiac injection [50, 51]. 4T1 cells express low levels of *Il13ra2* at baseline (Cq > 30). However, we found that 4T1 robustly upregulate *Il13ra2* in response to IL4 or IL13 (Fig. 1c). Given that IL4 and IL13 are abundant in tumors in vivo, and are already known to contribute to pro-metastatic signaling through type II IL4R [30–34], 4T1 provides an intriguing second model to investigate whether there are divergent effects when elevated IL13RA2 is environment-dependent rather than intrinsic.

### Characterization of IL13RA2-deficient models

To investigate the role of IL13RA2 in breast cancer metastasis, we generated two IL13RA2 CRISPR knockout models, MDA231BrM2-ΔIL13RA2 and 4T1-ΔIl13ra2. Efficient knockout of IL13RA2 was observed at the protein level in MDA231BrM2-ΔIL13RA2 by both flow cytometry and Western blot (Fig. 1d). Although we were unable to evaluate Il13ra2 protein levels in 4T1-ΔIl13ra2 due to lack of an effective antibody, qPCR demonstrated a significant decrease in *Il13ra2* transcript levels (Fig. 1e). Notably, the MDA231BrM2-ΔIL13RA2 cells showed a mild and variable increase in the other IL13 receptor, IL13RA1, while 4T1-ΔIl13ra2 cells show a decrease in *IL13RA1* transcript, suggesting that upregulation of *IL13RA1* is not a major compensatory mechanism (Online Resource 3, Fig. S1).

We then characterized the response of IL13RA2-proficient and -deficient cells to the type II cytokines IL4 and IL13, as a major recognized function of IL13RA2 is modulation of type II IL4 receptor signaling [36]. Neither MDA231BrM2-CTL nor MDA231BrM2-ΔIL13RA2 alter *IL13RA2* levels in response to IL4 or IL13 (Fig. 1f). 4T1-CTL cells, like unmodified 4T1, robustly upregulate *Il13ra2* in response to treatment with either IL4 or IL13. 4T1-ΔIl13ra2 fail to upregulate *Il13ra2* in response to either cytokine, supporting effective knockout (Fig. 1g).

### Loss of IL13RA2 confers a survival and growth advantage to TNBC cells in vitro

**Fig. 2:**
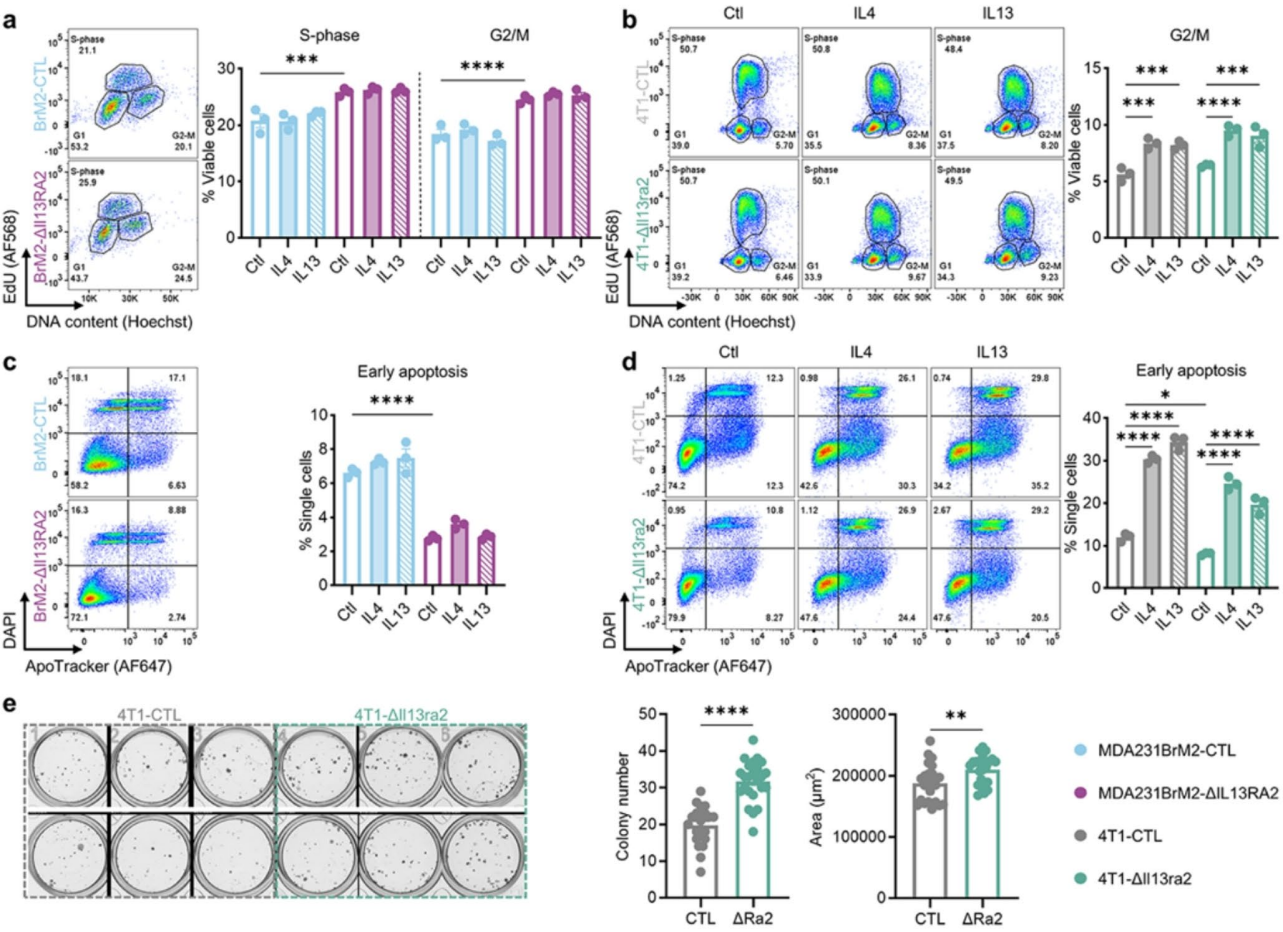
Loss of IL13RA2 confers a survival and growth advantage to TNBC cells in vitro. **a** Representative flow plots (left) and quantification (right) of the percentage of MDA231BrM2-CTL and ΔIL13RA2 in S-phase and G2/M by EdU/DNA-content stain after 72 h incubation in 2% media ± IL4 (10 ng/mL) or IL13 (20 ng/mL). Representative data (3 wells/group, *n* = 3 independent experiments). **b** Representative flow plots (left) and quantification (right) of the percentage of 4T1-CTL and ΔIl13ra2 in G2/M by EdU/DNA-content stain after 72 h incubation in 2% media ± IL4 (10 ng/mL) or IL13 (20 ng/mL). Representative data (3 wells/group, *n* = 3). **c** Representative flow plots (left) and quantification (right) of MDA231BrM2-CTL and ΔIL13RA2 in early apoptosis as measured by ApoTracker assay after 72 h incubation in 2% media ± IL4 (10 ng/mL) or IL13 (20 ng/mL). Representative data (3 wells/group, *n* = 3). **d** Representative flow plots (left) and quantification (right) of 4T1-CTL and ΔIl13ra2 in early apoptosis as measured by ApoTracker assay after 72 h incubation in 2% media ± IL4 (10 ng/mL) or IL13 (20 ng/mL). Representative data (3 wells/group, *n* = 3). In (**a–d**), statistical significance was determined by one-way ANOVA with Šídák’s multiple comparisons test with ****p < 0.0001, ***p < 0.001, and *p < 0.05. Non-significant comparisons omitted for clarity. **e** Comparison of colony formation by 4T1-CTL and 4T1-ΔIl13ra2. 200 cells/well seeded in 2% media and cultured for 6 days. Representative data shown; each dot represents an individual well (12–24 wells/group, *n* = 2 independent experiments). Statistical significance was determined by an unpaired *t*-test with ****p < 0.0001 and **p < 0.01. All error bars show SEM

We next characterized phenotypic differences of IL13RA2-deficient TNBC cells. Cell cycle analysis of MDA231BrM2-ΔIL13RA2 showed an elevated fraction of cells in S-phase and G2/M in comparison to MDA231BrM2-CTL (Fig. 2a). The basal proliferation of 4T1-ΔIl13ra2 is not significantly different to their control counterparts. Both 4T1-CTL and 4T1-ΔIl13ra2 show similarly increased proliferation in response to IL4 and IL13 (Fig. 2b).

We additionally investigated cell survival as a potential IL13RA2-dependent phenotype. MDA231BrM2-ΔIL13RA2 cells showed a significantly reduced fraction of cells in early apoptosis in comparison to MDA231BrM2-CTL, which was unaffected by cytokine stimulation (Fig. 2c). 4T1-ΔIl13ra2 also showed a reduced portion of cells in early apoptosis under basal conditions. While both 4T1-CTL and 4T1-ΔIl13ra2 demonstrated an elevated fraction of cells in early apoptosis following IL4/IL13 stimulation, the induction of cell death is blunted in Il13ra2-deficient cells, suggesting an overall survival advantage (Fig. 2d). In a clonogenic assay, 4T1-ΔIl13ra2 cells show increased colony number and size in comparison to 4T1-CTL (Fig. 2e), further supporting that Il13ra2 deficiency promotes survival and, to a lesser extent, proliferation. This clonogenic assay was not possible with the MDA231BrM2 cells due to their highly migratory nature that prevented the formation of single colonies.

### Loss of IL13RA2 increases aggressiveness of TNBC cells in vivo and correlates with reduced patient survival

**Fig. 3:**
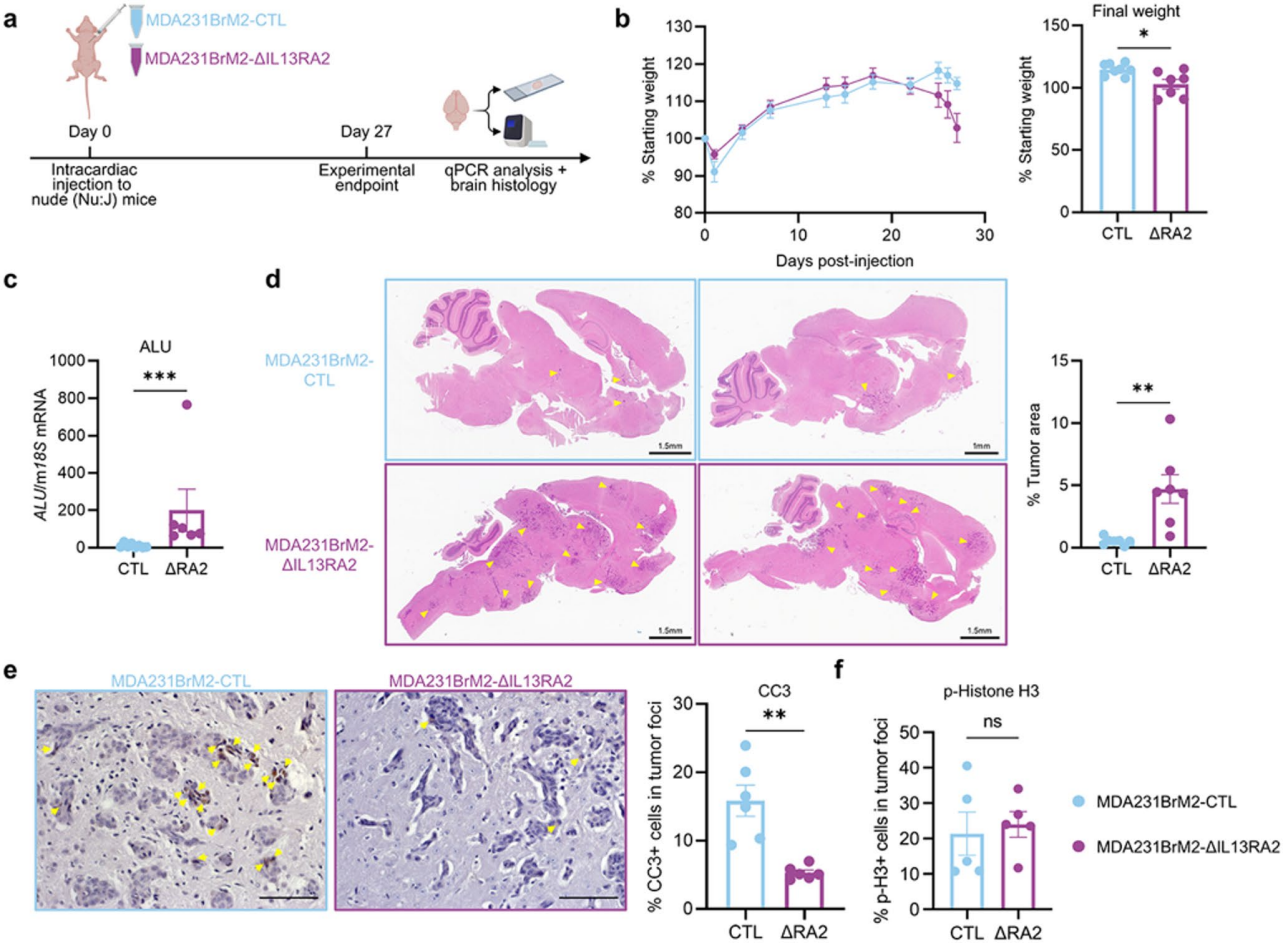
Tumor cell IL13RA2 deficiency increases mouse weight loss and brain metastatic burden following intracardiac injection. **a** Schematic of experimental workflow. **b** (Left) Comparison of animal weight loss (as percentage of starting weight) over time between MDA231BrM2-CTL and MDA231BrM2-ΔIL13RA2-injected animals (CTL: *n* = 8; ΔIL13RA2: *n* = 7). (Right) Comparison of animal weight loss (as percentage of starting weight) at the experimental endpoint (CTL: *n* = 8; ΔIL13RA2: *n* = 7). **c** Quantification of brain tumor burden at experimental endpoint by qPCR detection of human-specific *ALU* sequence (CTL: *n* = 8; ΔIL13RA2: *n* = 6). **d** (Left) Representative images of brain metastases in MDA231BrM2-injected mice; tumor foci are indicated with yellow arrowheads. Right, quantification of percent tumor area (CTL: *n* = 7; ΔIL13RA2: *n* = 7). **e** Representative images (left) and quantification (right) of cleaved caspase-3 (CC3) in MDA231BrM2-CTL and MDA231BrM2-ΔIL13RA2 brain metastases (*n* = 6 mice per group). CC3-positive cells are indicated with yellow arrowheads. **f** Quantification of phospho-histone H3 (p-H3) in MDA231BrM2-CTL and MDA231BrM2-ΔIL13RA2 brain metastases (*n* = 5 mice per group). Scale bars, 100 μm. Statistical significance was determined by Mann–Whitney tests with *p < 0.05, **p < 0.01, and ***p < 0.001. All error bars show SEM

To test the relevance of IL13RA2 in the metastatic setting, we performed intracardiac injection of MDA231BrM2-CTL or MDA231BrM2-ΔIL13RA2 cells in immunocompromised nude mice and tracked tumor development over four weeks (Fig. 3a). Animals that received ΔIL13RA2 cells lost significantly more weight at endpoint than their control-injected counterparts (Fig. 3b). The ΔIL13RA2-injected group showed increased brain tumor burden via qPCR detection of the human-specific ALU sequence (Fig. 3c). Histological analysis demonstrated that MDA231BrM2-ΔIL13RA2 injected animals had significantly increased tumor area (Fig. 3d). Concordant with the cancer cell survival advantage observed in vitro, MDA231BrM2-ΔIL13RA2 brain metastases showed significantly reduced cleaved caspase-3, an apoptotic marker, by immunohistochemistry (Fig. 3e). Immunohistochemical analysis of phospho-histone H3, an indicator of actively dividing cells, showed no difference in the tumor foci of mice injected with MDA231BrM2-CTL or MDA231BrM2-ΔIL13RA2 cells (Fig. 3f).

We also performed a pilot experiment with the 4T1-CTL and 4T1-ΔIl13ra2 cells in BALB/c mice. Of 4 mice receiving 4T1-ΔIl13ra2 cells, 3 did not survive to the 13-day endpoint, whereas the 4 mice receiving the 4T1-CTL cells did. The acute effects on survival severely limited our ability to collect tissue from 4T1-ΔIl13ra2-injected mice; therefore, histological assessments were not completed. Due to the aggressive nature of the model in vivo, we did not pursue it further.

To investigate whether these survival differences seen in our mouse models had clinical relevance, we assessed the relationship between overall survival and IL13RA2 levels in a cohort of patients with basal-like breast cancer (*n* = 309) using KM Plotter [49]. In this dataset, where the majority of patients had undergone some form of treatment, high expression of IL13RA2 in primary tumors was associated with improved patient survival (Online Resource 3, Fig. S2a). We also examined the link between IL13RA2 expression and initial diagnosis of brain/CNS metastases using data from the Metastatic Breast Cancer project (MBCproject.org). Although the number of patients diagnosed with brain metastases is very low, those with brain metastases at the time of metastatic diagnosis have significantly lower IL13RA2 levels than do those without metastases in the brain at diagnosis. This difference was not apparent in the setting of bone metastasis (Online Resource 3, Fig S2b).

### RNA-Seq identifies enrichment of survival-associated pathways in ΔIL13RA2 cells

We next performed bulk RNA-Sequencing (RNA-Seq) analysis to identify transcriptomic differences in IL13RA2-deficient cells. To facilitate identification of broadly applicable changes, we sequenced both untreated and IL13-treated 4T1-CTL and 4T1-ΔIl13ra2, as well as the human MDA231BrM2 pair. As 4T1 cells express very low levels of *Il13ra2* at baseline, the cytokine stimulated condition provided a more analogous model to the endogenously-high IL13RA2-expressing MDA231BrM2.

**Fig. 4:**
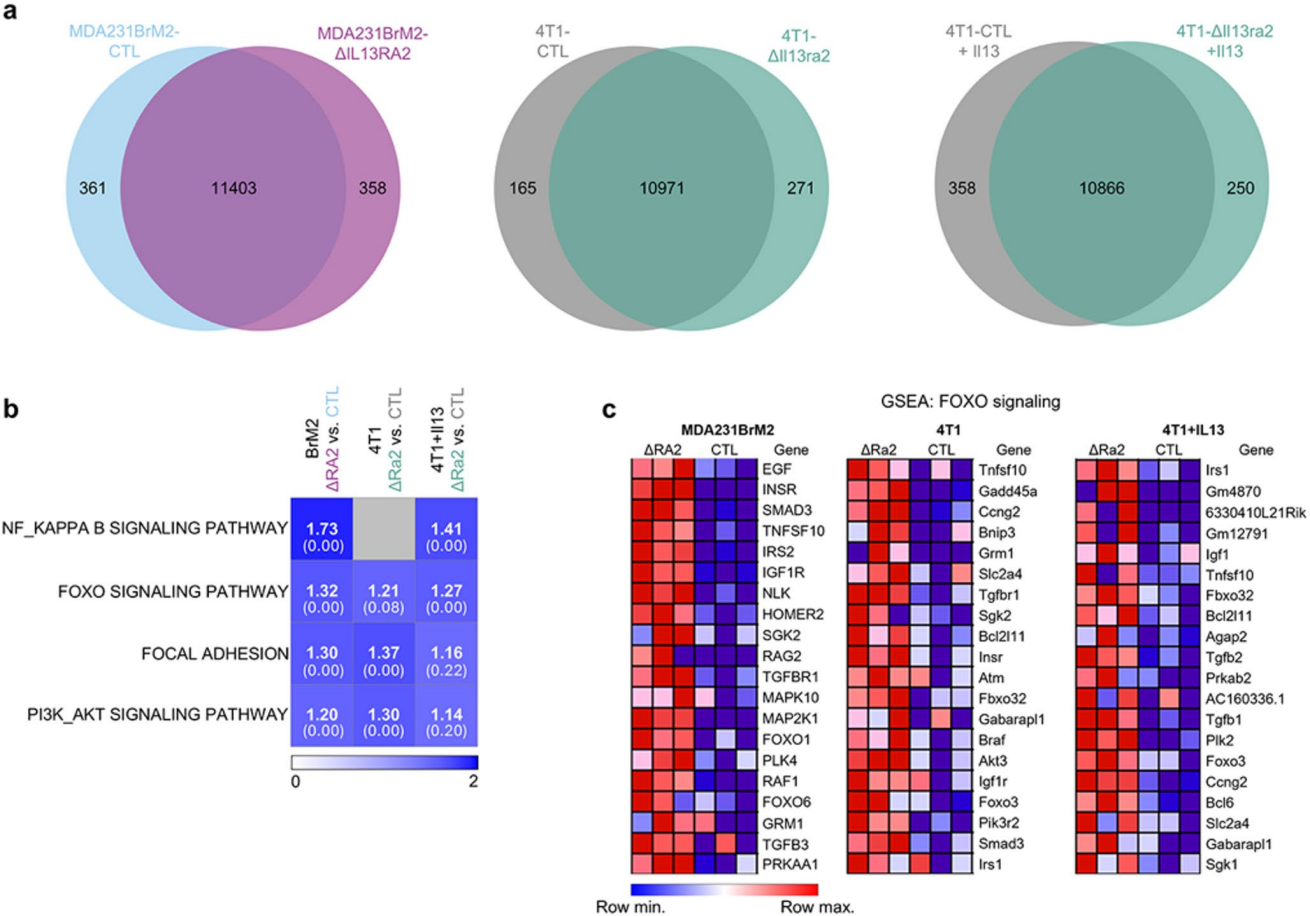
Bulk RNA-Seq identifies enrichment of survival-related pathways in IL13RA2-deficient cells. **a** A subset of genes is differentially expressed in ΔIL13RA2 cells as compared to their respective controls. **b** Heatmap showing normalized enrichment scores (NES) for the top 4 signaling pathways identified in ΔIL13RA2 cells across the three tested models (MDA231BrM2, 4T1 ± IL13) by gene set enrichment analysis (GSEA). The nominal p-value for each pathway is indicated in parentheses under the NES. Heatmap generated using Morpheus (https://software.broadinstitute.org/morpheus). Absolute color scale (0–2). **c** Heatmap showing relative gene enrichment levels for the top 20 genes in the FOXO pathway identified by GSEA in each dataset

Across the three models, loss of IL13RA2 impacted gene expression (Fig. 4a). We computed the average normalized enrichment score (NES) for each pathway across the three models and focused on the signaling pathways with the highest average NES score. The top four signaling pathways identified by GSEA were NF-κB, FOXO, focal adhesion, and PI3K-AKT (Fig. 4b). The FOXO pathway showed the most consistent enrichment amongst all three models, and the top twenty genes altered in this pathway are shown in Fig. 4c. We validated expression of a subset of highly altered genes in each cell line (Online Resource 3, Fig. S3a, b) and used these GSEA results to inform our mechanistic analysis.

### Lack of IL13RA2 results in changes to AKT and NF-κB signaling

We next focused on the mechanism underlying the observed survival/proliferation advantage of IL13RA2-deficient cells. Previous studies have identified CHI3L1 signaling through an IL13RA2/TMEM219 complex [41]. We therefore first asked if loss of IL13RA2 affected TMEM219 levels or CHI3L1-driven signaling. The CTL and IL13RA2-deficient cells expressed comparable levels of TMEM219 in both models (Online Resource 3, Fig. S4a, b). However, stimulation with CHI3L1 showed no effect on p-AKT (Online Resource 3, Fig. S4c) or p-ERK (Online Resource 3, Fig. S4d) in either MDA-MB-231 or MDA231BrM2-CTL. Thus, CHI3L1 does not appear to play a meaningful role in this model.

**Fig. 5:**
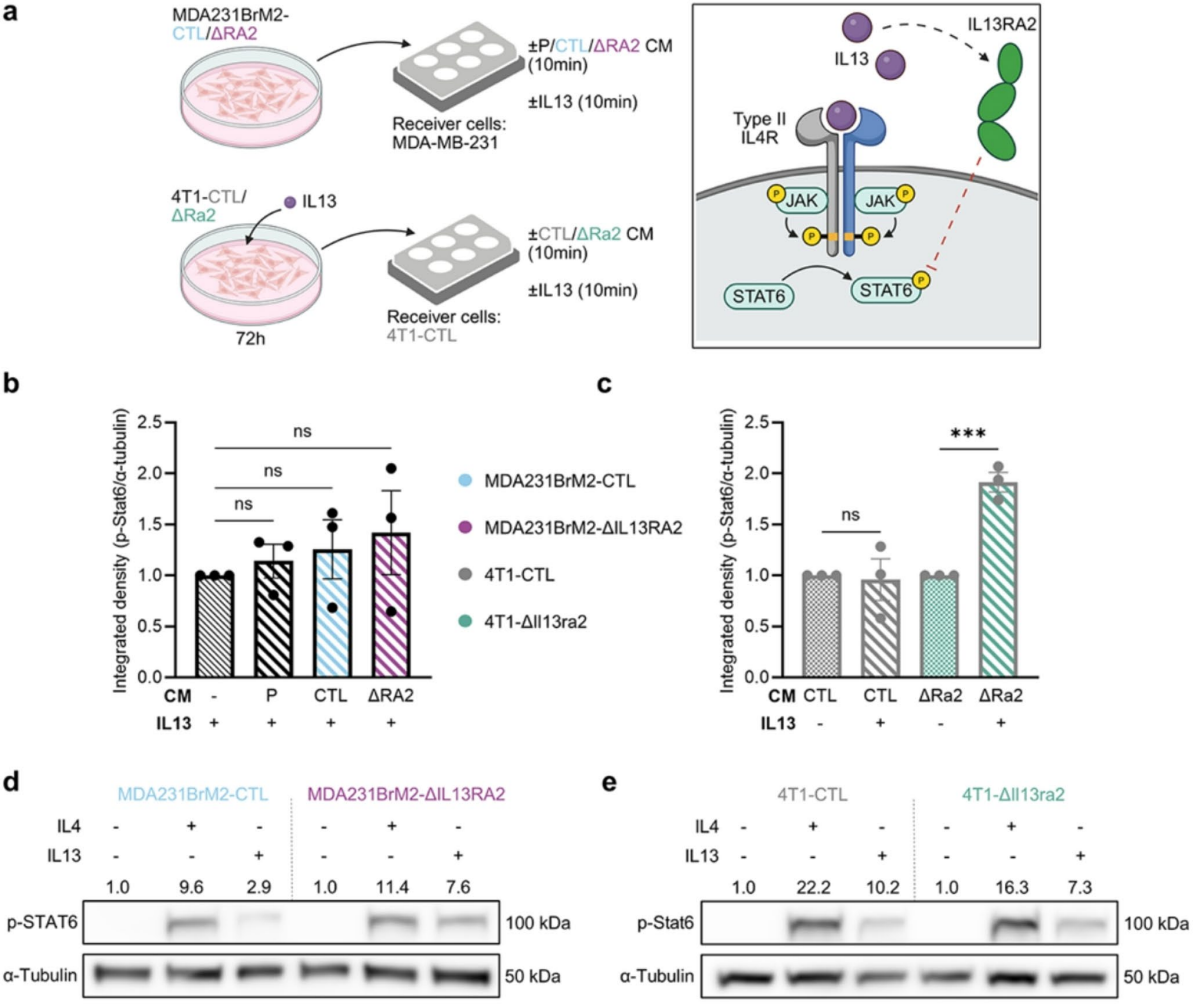
IL13RA2 has minimal decoy effect despite production of soluble decoy receptor by 4T1. **a** (Left) Schematic depicting experimental design to interrogate the contribution of soluble IL13RA2 to our system. CM, conditioned media. Lysates were collected and probed for p-STAT6 as a readout of IL13 signaling through type II IL4R. (Right) If soluble decoy IL13RA2 is present, IL13-induced p-STAT6 signal will be reduced. **b** Quantification of p-STAT6 signal intensity in MDA-MB-231 treated ± conditioned media taken from MDA231BrM2-CTL (CTL) or MDA231BrM2-ΔIL13RA2 (ΔRA2) for 10 min followed by IL13 (20ng/mL) for 10 min where indicated (*n* = 3). p-STAT6 normalized to α-tubulin and compared to signal strength in IL13-only condition. **c** Quantification of p-Stat6 signal intensity in 4T1-CTL treated ± conditioned media taken from IL13-treated 4T1-CTL (CTL) or 4T1-ΔIl13ra2 (ΔRa2) for 10 min followed by IL13 (20ng/mL) for 10 min where indicated (*n* = 3). p-Stat6 normalized to α-tubulin. Due to presence of residual IL13 in the conditioned media, signal strength in CM + IL13 condition is compared against the matching CM-only condition. **b, c** Statistical significance was determined by unpaired *t*-tests with ***p < 0.001 and ns, *p* > 0.05. Error bars show SEM. **d** p-STAT6 signal in IL4/IL13-treated MDA231BrM2-CTL and ΔIL13RA2. **e** p-Stat6 signal in IL4/IL13 treated 4T1-CTL and ΔIl13ra2. IL4, 10ng/mL; IL13, 20ng/mL; 20 min. p-STAT6 normalized to α-tubulin and compared to signal strength in respective untreated CTL. Representative blots (*n* = 3 per cell line)

The other frequently reported role of IL13RA2 is as a soluble decoy receptor [35, 36]. To probe whether our observed phenotypes were the result of a loss of soluble, decoy IL13RA2 and a resultant increase in IL13 signaling through the canonical type II IL4 receptor, we employed a media transfer experiment. We treated MDA-MB-231 cells, which are endogenously IL13RA2-negative, with conditioned media (CM) from MDA-MB-231, MDA231BrM2-CTL or MDA231BrM2-ΔIL13RA2 in the presence or absence of IL13 and measured STAT6 phosphorylation (see schematic, Fig. 5a). The 4T1 model required pre-treatment with IL13 to induce Il13ra2 expression in the CM-producing cells. If soluble IL13RA2 were a major product of any of these cell lines, we would expect reduced p-STAT6 in CM-exposed cells. MDA-MB-231 showed equivalent or increased p-STAT6 in presence of CM from all three cell lines (Fig. 5b), suggesting that soluble decoy IL13RA2 is not a major contributor in the human model. However, CM from Il13ra2-expressing 4T1 cells blunted IL13-mediated STAT6 phosphorylation, while CM from 4T1-ΔIl13ra2 failed to do so (Fig. 5c), suggesting that 4T1 may produce soluble decoy Il13ra2.

We next assayed signaling in response to IL4 or IL13 in standard culture conditions, beginning with the canonical downstream pathway of IL4/IL13 signaling, STAT6 [52]. MDA231BrM2-ΔIL13RA2 showed variable, but overall negligible alteration in IL13-induced p-STAT6 signal compared to CTL (Fig. 5d). In contrast to the CM results (Fig. 5c), 4T1-ΔIl13ra2 showed no change in p-Stat6 (Fig. 5e), suggesting that under standard culture conditions, the decoy effect of IL13RA2 is minimal in both models. Of note, Márquez-Ortiz [16] described an ephrin-dependent (EFNB1/EPHB1) mechanism underlying an IL13RA2 function in breast cancer brain metastasis. While *EFNB1* showed a mild (< 1.5-fold) increase in MDA231BrM2-ΔIL13RA2, *EPHB1* expression was almost undetectable in either cell line (Online Resource 3, Fig. S3c), suggesting that this pathway is not a major contributor in our model. Examination of RNA-seq data for the 4T1 cells indicate no change in *Efnb1* expression associated with *Il13ra2* deficiency, while *Ephb1* is completely absent.

**Fig. 6:**
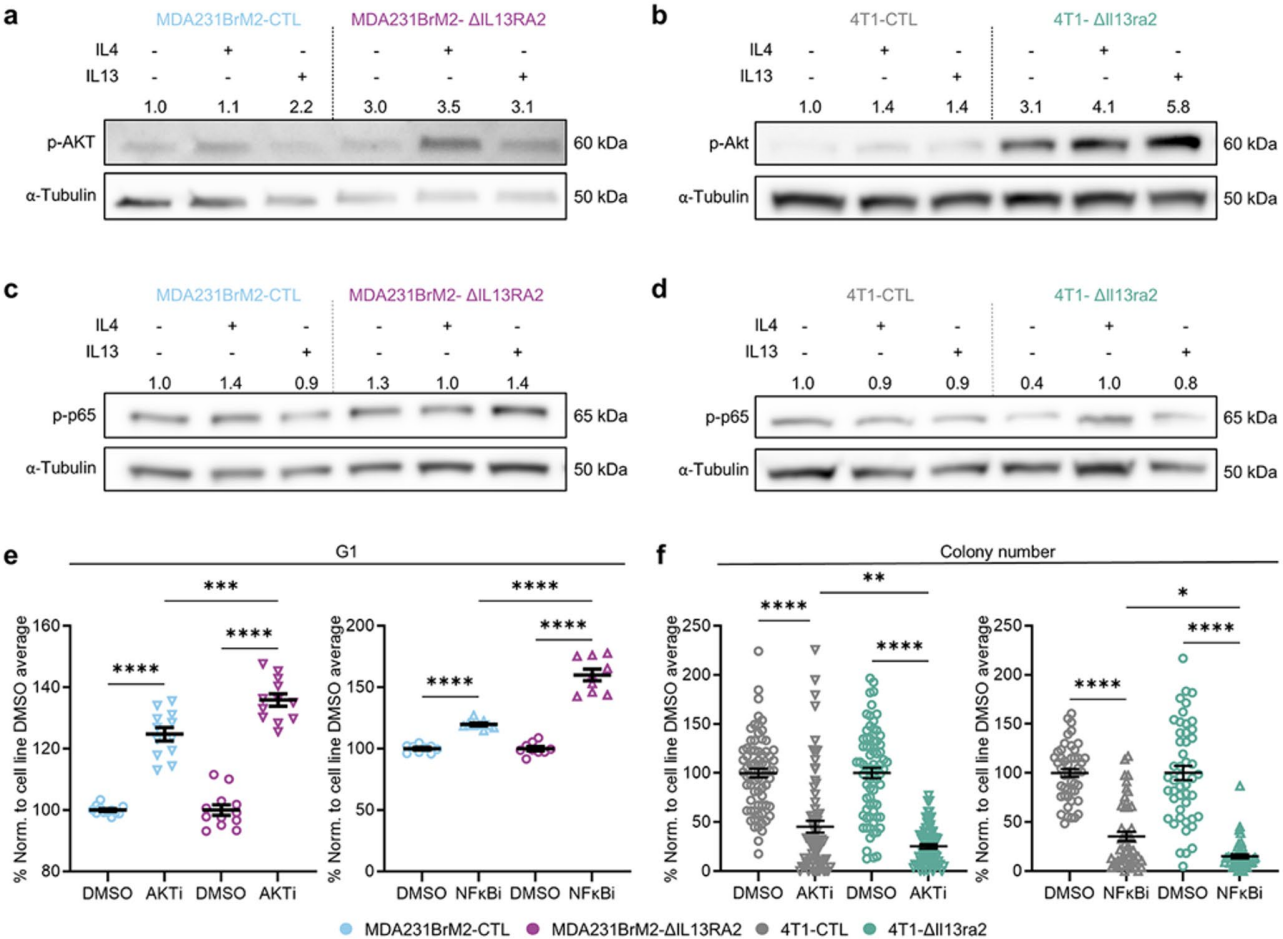
NF-κB and AKT signaling is altered in IL13RA2-deficient cells. **a** p-AKT signal in IL4/IL13-treated MDA231BrM2-CTL and ΔIL13RA2. Global image maxima of p-AKT blots were decreased to visualize bands for quantification due to faint signal; blot used for quantification shown. Normalized band intensities are shown above each lane. Representative blots (*n* = 3). **b** p-AKT signal in IL4/IL13-treated 4T1-CTL and ΔIl13ra2. Global image maxima of p-AKT blots were decreased to visualize bands for quantification due to faint signal; quantified images shown. Normalized band intensities are shown above each lane. Representative blots (*n* = 3). **c** p-p65 signal in IL4/IL13-treated MDA231BrM2-CTL and ΔIL13RA2. Normalized band intensities are shown above each lane. Representative blots (*n* = 2). **d** p-p65 signal in IL4/IL13-treated 4T1-CTL and ΔIl13ra2. Normalized band intensities are shown above each lane. Representative blots (*n* = 2). **a–d** IL4, 10ng/mL; IL13, 20ng/mL, 20 min. Band intensity normalized to α-tubulin and compared to signal strength in respective untreated CTL line. **e** Percentage of cells in G1 by EdU/DNA content stain as normalized to the average of the corresponding cell line vehicle control after 72 h exposure to ipatasertib (AKTi,15µM), BMS-345,541 (NF-κBi, 1µM), or DMSO (vehicle). Each point represents an individual well (3 wells/group; ipatasertib, *n* = 4 independent experiments; BMS-345541, *n* = 3). **f** Colony formation by 4T1-CTL and 4T1-ΔIl13ra2 in presence of ipatasertib (AKTi, 2.5µM), BMS-345,541 (NF-κBi, 1µM), or DMSO (vehicle). Data normalized to average of the corresponding cell line vehicle control. 200 cells/well seeded in 2% media and cultured for 6 days. Each point represents an individual well (24 wells/group; ipatasertib, *n* = 3; BMS-345541, *n* = 2). **e, f** Statistical significance was determined by one-way ANOVA with Šídák’s multiple comparisons test with ****p < 0.0001, ***p < 0.001, **p < 0.01, *p < 0.05. All error bars show SEM

In agreement with the enrichment of AKT-related signaling by GSEA analysis, we observed a substantial increase in baseline p-AKT in both ΔIL13RA2 cell lines (Fig. 6a, b). Additionally, in the 4T1 model, ΔIl13ra2 cells showed increased induction of p-Akt in response to cytokine (Fig. 6b).

We also investigated alterations in NF-κB signaling due to the enrichment of this pathway by GSEA analysis. We observed a moderate increase in phosphorylated p65 (p-p65) in MDA231BrM2-ΔIL13RA2 as compared to control. This increase was unaffected by treatment with IL4/IL13 (Fig. 6c). In contrast, 4T1-ΔIl13ra2 showed reduced p-p65 at baseline, but enhanced responsiveness to IL4/IL13 relative to the untreated condition (Fig. 6d).

To establish whether these signaling alterations have a causal relationship with the survival and growth phenotype of ΔIL13RA2 cells, we evaluated the response of CTL and ΔRA2 cells to ipatasertib, a pan-AKT inhibitor, or BMS-345,541, an NF-κB pathway inhibitor. As MDA231BrM2 cells are too migratory to obtain clonogenic results, we used cell-cycle analysis to evaluate response. Following treatment, we observed an accumulation of cells in G1 in both MDA231BrM2-CTL and ΔIL13RA2, indicating cell cycle arrest. MDA231BrM2-ΔIL13RA2 was significantly more sensitive to either drug than their CTL counterpart (Fig. 6e). Clonogenic assays in 4T1-CTL and 4T1-ΔIl13ra2 revealed analogous phenotypes. Treatment provoked a decrease in colony number, indicative of reduced cell survival, in both cell lines, but 4T1-ΔIl13ra2 showed a significantly greater decrease than did CTL cells (Fig. 6f). In addition, we performed a Cyquant assay, which assesses levels of nuclear DNA as a proxy for cell number, after treatment with the inhibitors. In both cell lines, the IL13RA2-deficient cells had significantly lower signal, indicative of lower cell number, than their CTL counterparts after treatment with ipatasertib. In this assay, the NF-kB inhibitor did not have a similar effect (Online Resource 3, Fig. S5). Taken together, these data establish a causal link between enhancement of AKT and NF-κB signaling and improved survival in IL13RA2-deficient cells. Further, the dramatic sensitization of IL13RA2-deficient cells to inhibition of either pathway, but especially AKT, may represent a therapeutically useful vulnerability.

## Discussion

In this study, we demonstrate that knockout of IL13RA2 confers an unexpected benefit to TNBC cells. In two distinct models, IL13RA2-deficient cells demonstrate improved cell survival and, to a lesser extent, proliferation in vitro, as well as a notable increase in brain metastatic capacity and reduced apoptosis in vivo. Further, we demonstrate that these phenotypes are mediated through AKT and NF-κB and that loss of IL13RA2 sensitizes cells to pharmacologic inhibition, particularly of AKT.

IL13RA2 was first of interest because of its elevated, tumor-specific expression pattern in gliomas [53]. This led to development of a possible targeted treatment, IL13 conjugated to pseudomonas exotoxin (IL13-PE), which potently and specifically killed glioblastoma (GBM) cells [11]. As of April 2025, there are 7 registered clinical trials using IL13RA2-targeted agents as anticancer therapy (trial IDs: NCT05168423, NCT06355908, NCT04003649, NCT06186401, NCT04119024, NCT02208362, NCT06815029; [54]). Though generally well-tolerated, clinical success has been modest [43]. Only IL13-PE progressed to a phase III trial, where it failed to show benefit beyond standard of care [55]. In addition to utilizing IL13RA2 as a tumor-specific target, there is interest in blocking IL13RA2 signaling itself [46]. IL13RA2 inhibition has resulted in tumor regression in murine models of glioma and colorectal cancer [44, 45, 56]. However, our data suggest this would be deleterious in TNBC, highlighting the importance of considering specific tumor context for targeted therapies.

IL13RA2 has a pro-tumorigenic function in some contexts, notably brain and colorectal cancers [17–21, 57, 58], and to a lesser extent pancreatic [38, 40] and ovarian [39] cancers, but the overall relationship between IL13RA2 expression and patient prognosis remains ambiguous. A recent study in GBM found that high levels of IL13RA2 in patient plasma or tumor samples predicted improved survival [25]. The authors note that their study evaluated IL13RA2 protein expression, while Han et al. [58] evaluated transcript expression, also in GBM, and reported contradictory results. Many other studies (e.g [17, 22, 57])., reporting a correlation between high IL13RA2 levels and poor prognosis in primary brain tumors also use gene expression data. It is possible that the correlation between IL13RA2 and patient survival may differ between transcript and protein. Interestingly, we observed complete knockout of IL13RA2 at the protein level in MDA231BrM2-ΔIL13RA2 cells. However, *IL13RA2* transcript was slightly higher than in CTL cells. Our data together with previous clinical studies suggest that the concordance between protein and mRNA levels of IL13RA2 may vary.

Our results agree with other studies relating low IL13RA2 levels to pro-tumorigenic effects. One study demonstrated that overexpression of IL13RA2 impaired tumor growth in murine models of breast and pancreatic cancer [27]. In hepatocellular carcinoma, elevated IL13RA2 correlated with improved patient survival, while silencing IL13RA2 had pro-proliferative, pro-survival effects analogous to those reported here, albeit via ERK activation [24]. Interestingly, reduced IL13RA2 expression was also identified in keloid fibroblasts, which are highly proliferative compared to normal skin. IL13RA2 knockdown in normal human fibroblasts induces a keloidal phenotype marked by increased proliferation and apoptotic resistance [59]. In keloids, the pro-proliferative effects occur via the decoy effect of IL13RA2 and reduced IL13RA2 leads to accelerated IL13-induced fibrosis through STAT6. Overall, beneficial or detrimental functions of IL13RA2 appear to be dependent on tissue type.

Márquez-Ortiz et al. [16] previously examined the role of IL13RA2 in breast cancer brain metastasis. Unlike our results, they found that IL13RA2 expression promoted proliferation in human TNBC and HER2 + breast cancer cells while downregulation impaired brain metastasis. Importantly, that study employed shRNA-mediated knockdown of *IL13RA2*, and the authors reported that full receptor knockout resulted in cell death. In contrast, we generated CRISPR knockouts of IL13RA2 in both MDA231BrM2 and 4T1.The difference in lethality of IL13RA2 knockout may be attributed to a difference in the cells used (our MDA231-BrM2 are from a distinct source compared to Márquez-Ortiz), or to an important difference in methodology as we pooled CRISPR guides and obtained a non-clonal knockout population by cell sorting rather than selecting individual IL13RA2 knockout clones. Further, the Marquez-Ortiz studies utilized a different type of immunodeficient mice than we did (NSG versus nude) for the in vivo studies with human cell lines that could have resulted in differential immune cell interactions. However, we also used immunocompetent BALB/c mice for in vivo studies with 4T1 cells; though those studies were limited by the aggressive nature of the model, the poor survival of animals receiving Il13ra2-deficient tumor cells suggests that immune interactions are not a critical modifier of the results. Ultimately, it is likely that the divergence of our observations arises from the difference between partial and near-complete loss of IL13RA2, as well as in EphrinB1, which was a critical player in their study but absent in our cells.

While the complete signaling network of IL13RA2 remains to be elucidated, several pathways have been identified. IL13/IL13RA2 interactions result in activation of AP-1 [37–40] and Src-related pathways [18–21], leading to TGFβ upregulation [37] and increased PI3K/AKT and ERK signaling [18–21, 45, 60]. The Casal group has identified multiple binding and signaling partners for IL13RA2. In colon cancer, they determined the scaffolding protein FAM120A to be an essential mediator of IL13/IL13RA2 signaling through FAK, PI3K and Src [19]. In GBM, protein tyrosine phosphatase 1B (PTPB1) interacts directly with IL13RA2 to mediate IL13-induced Src activation [20], resulting in activation of the transcription factor Schnurri3 and promotion of proliferation and invasion via WNT, ERK, and MMP9 [21]. Others have identified diverse additional functions of IL13RA2. In Hermansky-Pudlak Syndrome lung disease [61], galectin-3 competes directly with TMEM219 to bind IL13RA2. Another study found that IL13RA2 physically associates with p53 and its E3 ligase, UBE3C, in colon cancer [62]. Two reports, in glioblastoma [63] and lung epithelial cells [64], showed interactions between the cytoplasmic domains of IL13RA2 and IL4R that interfere with IL4-driven STAT6 signaling. Finally, IL13RA2 interacts with EGFRvIII, but not wild-type EGFR, in GBM to promote MAPK, AKT, and STAT3 signaling in a cytokine-independent manner [17].

Taken together, it is clear that IL13RA2 has highly heterogeneous signaling roles. Though our phenotypic data contradict those reported by the Casal group for colon cancer [18, 19, 45, 46], the pathways identified as differentially active in IL13RA2-deficient cells, namely, AKT, NF-κB, and focal adhesion, overlap. A diversity of binding/signaling partners have been identified for IL13RA2, including FAM120A [19], which is a scaffold for many other proteins. Future studies should therefore consider other, as-yet-unidentified proteins interacting with IL13RA2 that result in inhibitory signaling.

We also cannot exclude the possibility that our observed effects are mediated through loss of the decoy action of IL13RA2, similarly to keloidal signaling [59]. However, we observed a decoy effect only when IL13RA2 was present at extraordinarily high levels, such as concentrated conditioned media from IL13-treated 4T1-CTL. Under normal culture conditions, the negligible alteration in p-STAT6 response and the sensitivity of our IL13RA2-deficient cells to AKT and NF-κB inhibition in absence of cytokine stimulation argue against loss of the decoy as the primary mechanism and support the many studies highlighting a signaling role for this receptor.

A critical remaining question is why IL13RA2 expression is elevated in many metastatic models, including MDA231BrM2, if loss of the receptor is ultimately beneficial to brain metastatic cells. Kawakami et al. [27], who observed growth inhibition by IL13RA2 overexpression in a breast cancer model, noted that IL13RA2 levels decrease over time in vivo, suggesting that IL13RA2 deficiency is beneficial during the later stages of tumor growth. Of note, many groups reported that IL13RA2 expression correlates with elevated invasive or migratory potential [18, 20, 38, 39, 45, 56] while we observe a strong pro-survival phenotype in IL13RA2-deficient cells. Together, these findings suggest that IL13RA2 may be pro-tumorigenic in the early stages of colonization in brain metastatic TNBC through promoting an invasive phenotype. However, cells that downregulate IL13RA2 may be more successful in metastatic outgrowth through enhanced survival signaling.

Limitations of our study include the inability to confirm 4T1 Il13ra2 status at the protein level, lack of histological analysis for the 4T1-injected mice due to unexpected animal deaths, and a lack of diverse cell line models. Future studies with additional cell lines but more critically, endogenous in vivo models that demonstrate spontaneous brain metastasis, will provide useful insights. However, we generated robust CRISPR knockouts of IL13RA2 derived from multiclonal populations, with thorough validation of observations across two TNBC models using multiple orthogonal methods. Further, MDA231BrM2-ΔIL13RA2 produce robust and widespread metastatic lesions in the brain, which may offer an improved model for studying brain metastasis. One further caveat of the work shown here is that the human survival data shown in Online Resource 3, Fig. S2a is not corrected for any potential differences in patient age or treatment duration as this information was not available from the public resource used. Further studies in additional patient cohorts will be needed to better delineate the role and prognostic value of IL13RA2 in patients with breast cancer brain metastases.

In conclusion, this study indicates that IL13RA2 deficiency promotes tumor cell survival, growth, and metastasis in models of brain-metastatic TNBC. Our data suggest that IL13RA2 inhibition is unlikely to benefit patients with TNBC, although those with IL13RA2-low tumors may benefit from AKT inhibitors. Future studies should focus on elucidating the signaling partners of IL13RA2 in TNBC, identifying other contexts where IL13RA2 deficiency is pro-tumorigenic, and investigating the potential of a reciprocal relationship between IL13RA2 and the type II IL4 receptor beyond the decoy paradigm.

## Electronic supplementary material

Below is the link to the electronic supplementary material.

Supplementary Material 1

Supplementary Material 2

Supplementary Material 3

## Data Availability

Raw and processed RNA-Seq data were deposited in the Gene Expression Omnibus (GEO) under project number GSE290037 and are available at the following URL: https://www.ncbi.nlm.nih.gov/geo/query/acc.cgi?acc=GSE290037. All other data supporting the findings of this study are included in the paper and its supplementary information.
