# Supplementary material for: Loss of IL13RA2 promotes metastatic tumor growth in triple-negative breast cancer via increased AKT and NF-κB signaling
Source: Clin Exp Metastasis. 2025 Jul 15;42(5):40. doi: 10.1007/s10585-025-10362-1 (PMC12263738; doi:10.1007/s10585-025-10362-1)
Supplement: Supplementary file 3 — Supplementary Material 3 [file 10585_2025_10362_MOESM3_ESM.pdf]

Online Resource 3: Supplemental Figures

Article title: Loss of IL13RA2 promotes metastatic tumor growth in triple-negative breast cancer via increased AKT and NF-κB signaling

Journal: Clinical & Experimental Metastasis

Authors: Wendy E Bindeman, Kevin C Corn, Marjan Rafat, and Barbara Fingleton*

*Corresponding author information:

Email: barbara.fingleton@vanderbilt.edu

Affiliation: Department of Pharmacology, Vanderbilt University, Nashville, TN, USA

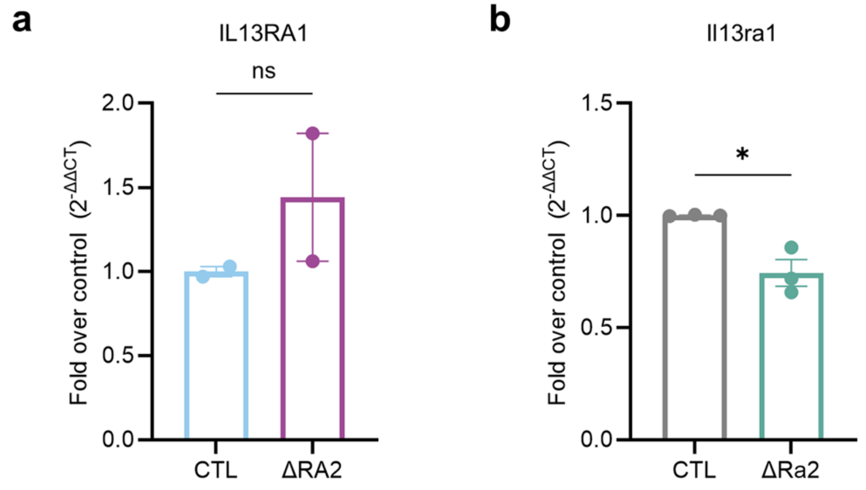

Fig. S1 IL13RA2-deficient cells do not upregulate *IL13RA1*. **a** Comparison of *IL13RA1* transcript levels in MDA231BrM2-CTL and MDA231BrM2-ΔIL13RA2 (n=2). **b** Comparison of *Il13ra1* transcript levels in 4T1-CTL and 4T1-ΔIl13ra2 (n=3). (**a-b**) Statistical significance was determined by unpaired t-tests with *$p<0.05$ and non-significant (ns) $p>0.05$. All error bars show SEM.

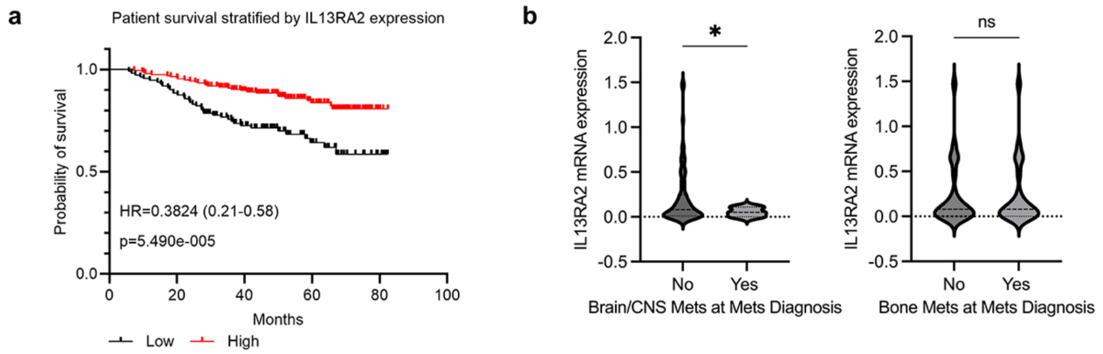

Fig. S2 Low IL13RA2 expression associates with poor prognosis and/or brain metastases in breast cancer. a Patient prognosis as a function of primary tumor *IL13RA2* expression in a cohort of patients with basal-like breast cancer. Data from KM Plotter Breast Cancer RNA-Seq database[49]. N=309 (low=112; high=197). Expression cutoff value automatically selected by KM Plotter; expression threshold=0.77, false discovery rate 1%. Statistical significance was determined by log-rank (Mantel-Cox) test. **b** Patients with metastatic breast cancer who are diagnosed with brain/CNS metastases at the time of metastatic disease diagnosis have a significantly lower expression level of IL13RA2 compared to those without brain metastases (No, N=80; Yes, N=3). There is no difference for patients who are or are not diagnosed with bone metastases at the time of metastatic disease diagnosis (No, n=26; yes, n=26). Mets, metastases. Data from the Metastatic Breast Cancer Project (accessed via CBioPortal, 15 June 2025). Statistical significance was determined by t-test with Welch's correction with *$p<0.05$ and non-significant (ns) $p>0.05$

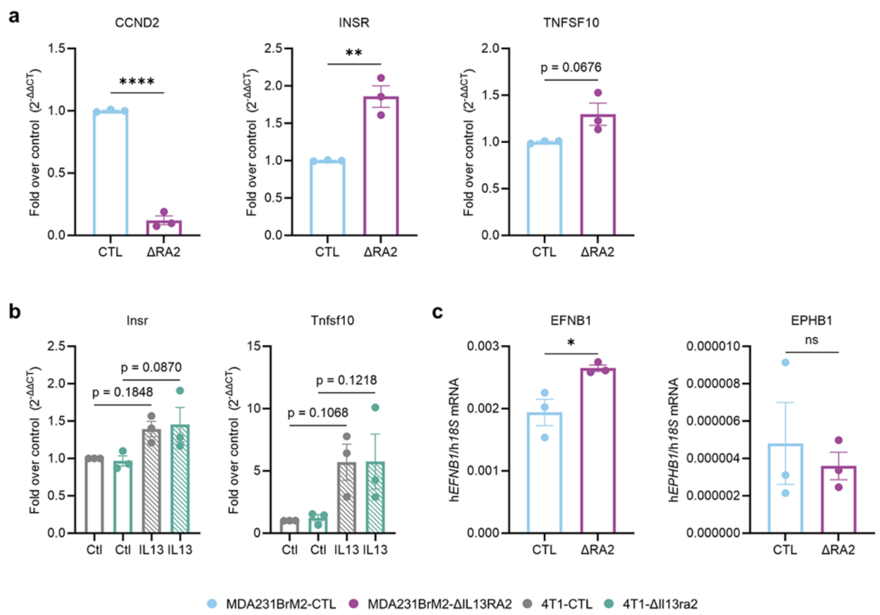

Fig. S3 Validation of differentially regulated genes identified by RNA-Seq. **a** Validation of selected differentially regulated genes in MDA231BrM2-CTL and ΔIL13RA2 (n=3). Statistical significance was determined by unpaired t-tests with ****p<0.0001 and **p<0.01. **b** Validation of selected differentially regulated genes in 4T1-CTL and ΔIl13ra2 (n=3). Statistical significance was determined by one-way ANOVA with Šídák's multiple comparisons tests; only p<0.2 shown. **c** *EFNB1* and *EPHB1* gene expression in MDA231BrM2-CTL and ΔIL13RA2. Data shown as unnormalized averages from three independent experiments. Statistical significance was determined by unpaired t-tests with *p<0.05 and non-significant (ns) p>0.05. Data in (**c**) shown as fold change relative to housekeeping gene ($2^{-\Delta CT}$) to demonstrate near-absence of *EPHB1* expression. All others shown as fold change relative to control ($2^{-\Delta\Delta CT}$). All error bars show SEM

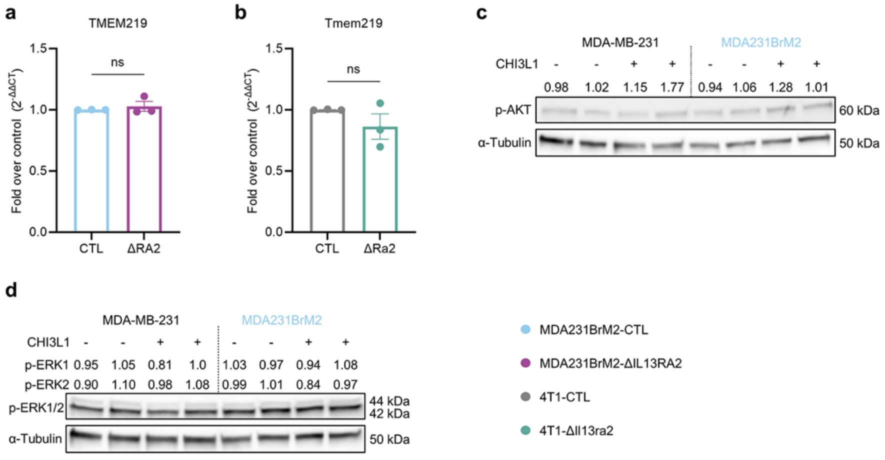

Fig. S4 TMEM219 and CHI3L1 are not major contributors to IL13RA2-dependent signaling in our models. **a** Comparison of *TMEM219* gene expression in MDA231BrM2-CTL and MDA231BrM2-ΔIL13RA2 (n=3). **b** Comparison of *Tmem219* gene expression in 4T1-CTL and 4T1-ΔIl13ra2 (n=3). (**a-b**) Statistical significance was determined by unpaired t-test with non-significant (ns) p>0.05. Error bars show SEM. **c** p-AKT signal in MDA-MB-231 and MDA231BrM2 following CHI3L1 treatment. Global image maxima of p-AKT blots were decreased to visualize bands for quantification due to faint signal; quantified images shown. **d** p-ERK1/2 signal in MDA-MB-231 and MDA231BrM2 following CHI3L1 treatment. CHI3L1, 500ng/mL; 30min (n=1). (**c-d**) Relative band intensity normalized to α-tubulin and compared to the average of the corresponding cell line control. Experiments performed using unmodified MDA231BrM2

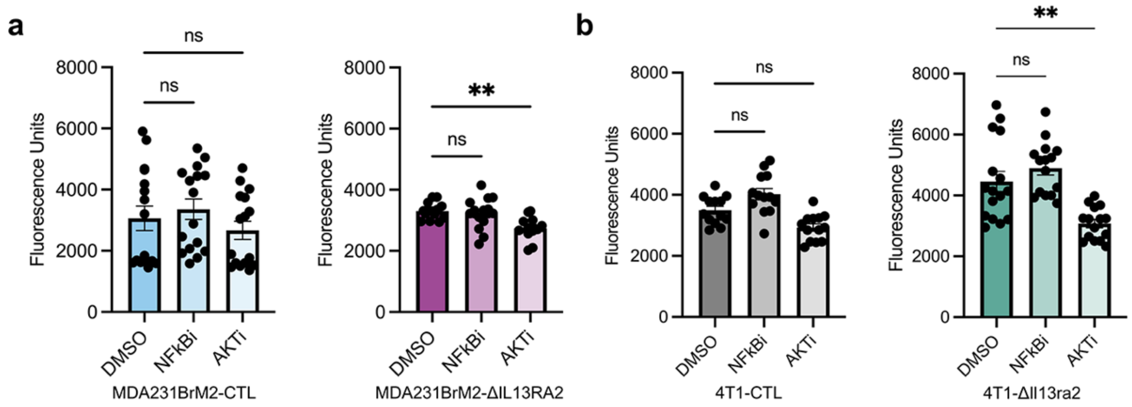

Fig. S5 The AKT inhibitor ipatasertib significantly reduces cell number of IL13RA2-deficient but not control cells as determined by Cyquant assay in both (**a**) MDA231BrM2 and (**b**) 4T1 cells. Cells were exposed to ipatasertib (AKTi,15μM), BMS-345541 (NF-κBi, 1μM), or DMSO (vehicle) for 72h and analyzed at endpoint using the Cyquant Direct assay (n=8-15 wells per condition). Statistical significance was determined by Kruskal-Wallis with Dunn's multiple comparisons test with **$p<0.01$ and non-significant (ns) $p>0.05$. Data shown are representative of 2 independent experiments. All error bars show SEM
